# Altered glutamatergic tone reveals two distinct resting state networks at the cellular level in hippocampal sclerosis

**DOI:** 10.1038/s41598-017-00358-7

**Published:** 2017-03-23

**Authors:** Jyotirmoy Banerjee, Aparna BanerjeeDixit, Arpna Srivastava, Bhargavi Ramanujam, Aanchal Kakkar, Chitra Sarkar, Manjari Tripathi, P. Sarat Chandra

**Affiliations:** 1 Centre of Excellence for Epilepsy, National Brain Research Centre, Manesar, India; 2 Department of Neurosurgery, All India Institute of Medical Sciences, New Delhi, India; 3 Department of Neurology, All India Institute of Medical Sciences, New Delhi, India; 4 Department of Neuropathology, All India Institute of Medical Sciences, New Delhi, India

## Abstract

Hippocampal sclerosis (HS), the most common subset of drug-resistant epilepsy (DRE), is associated with large-scale network abnormalities, even under resting state. We studied the excitatory postsynaptic currents (EPSCs) recorded from pyramidal neurons in resected samples under resting conditions from the hippocampal and anterior temporal lobe (ATL) obtained from patients with HS (n = 14) undergoing resective surgery. We observed higher frequency and amplitude of spontaneous EPSCs in both the samples compared to non-seizure control samples. Application of tetrodotoxin (TTX) reduced the frequency of spontaneous EPSCs by 49.6 ± 4.3% and 61.8 ± 6.2% in the hippocampal and ATL samples, respectively. The magnitude of reduction caused by TTX with respect to non-seizure controls was significantly higher in the ATL samples than in the hippocampal samples. The magnitude of the change in the expression of the NR2A subunit of the NMDA receptors also varied in these two regions. Thus, the mechanism of hyperexcitabilty mediated by glutamatergic network reorganization in the hippocampal region is different from that in the ATL region of patients with HS, suggesting two independent resting-state networks at the cellular level. Taken together, these findings will improve the understanding of the broadly distributed resting-state networks in HS.

## Introduction

The most common form of drug-resistant epilepsy (DRE) is hippocampal sclerosis (HS), where the mesial temporal lobe structures (including the hippocampus, amygdala and other enterorhinal structures) are involved in seizure generation through abnormal neuronal networks^1^. Currently, the surgical procedures used for the treatment of HS include standard anterior temporal lobectomy, combined with amygdalo-hippocampectomy and selective amygdalo-hippocampectomy (SAH). The procedures also involve removal of the uncus, para-hippocampal gyrus, enterorhinal cortex, and subiculum, specifically in anterior temporal lobectomy^2–6^. A systematic review and meta-analysis of the literature suggests that anterior temporal lobectomy may have a better outcome compared with SAH, suggesting that a part of the abnormal network could also be situated in extra-hippocampal regions^2^.

HS is a large-scale distributed network disorder and may not only involve the hippocampus but also extra-hippocampal areas^7^. Depth electrode studies of HS suggest that in addition to the hippocampus, sites of ictal onset and immediate spread could also involve two or more mesial-temporal, lateral-temporal, and/or extra–temporal structures^8^. Ictal depth electroencephalography (EEG) and magnetic resonance imaging (MRI) studies have suggested two epileptogenic networks in patients with HS where both the hippocampus and extra-hippocampal regions are involved^9^. Functional MRI (fMRI) studies have suggested that in temporal lobe epilepsy, even the spontaneous activity of networks is affected^10^. More recently it has been shown in patients with HS that alteration in electrophysiologic functional hubs leads to pathophysiologic brain network reorganization, even under resting-state^11^. Dysfunctional resting state networks have been reported not only in the hippocampal region but also in the extra-hippocampal structures in patients with HS^10^. Although resting-state networks have been reported in HS, the underlying cellular mechanisms responsible for generation of multiple networks still remain poorly understood. Moreover, it is also unclear whether these networks are independent of each other. Alteration at the synaptic transmission level under resting conditions can lead to the generation of network hubs in these regions.

Excessive glutamatergic response in the epileptogenic foci has been proposed to be an important mediator of hyperexcitability in HS. Previously, we showed that under resting conditions, spontaneous EPSCs on pyramidal neurons were higher in resected hippocampal samples obtained from patients with HS^12^, suggesting a glutamatergic network reorganization in the hippocampus. However, it is not known if the extent of change in excitatory synaptic transmission is similar in the extra-hippocampal regions of patients with HS. We have earlier reported that under resting conditions, endogenous NMDA receptor activity was enhanced in the hippocampal samples obtained from patients with HS^12^. The differential role of NR2A and NR2B subunits carrying NMDA receptors in epileptogenesis has also been reported in animal models of HS^13–16^, suggesting increased excitability due to altered properties of NMDA receptor subunits. A study on hippocampal and temporal cortical samples obtained from patients with temporal lobe epilepsy suggested that a splice variant of the NR1 subunit did not significantly contribute to the pathophysiology^17^. However, the role of NR2A and NR2B subunits carrying NMDA receptors associated with glutamatergic activity in a region-specific manner in epileptic brain tissues in HS is still not known.

Thus, we utilized cellular and molecular strategies to determine the configuration of the glutamatergic networks under resting state, particularly to ascertain whether there is a single hippocampal related network or whether there are other independent networks in the extra-hippocampal regions that may contribute toward seizure generation. The targeted tissues resected from the hippocampal and anterior temporal lobes (ATL) of patients with HS were used for cellular electrophysiological analysis. The glutamatergic input on to pyramidal neurons of the hippocampal and ATL was investigated, and this activity was compared between both regions (Hippocampus and ATL) as well as with samples of brain resected from non-seizure controls. To further examine at the molecular level alterations that may lead to the generation of abnormal networks, we analyzed the mRNA levels of the NR2A and NR2B subunits using quantitative PCR and quantified the protein levels of NR2A and NR2B using western blotting analyses in both regions.

## Results

### Histopathology and immunohistochemistry revealed typical hippocampal sclerosis but no neuronal loss and gliosis in ATL samples

**Figure 1:**
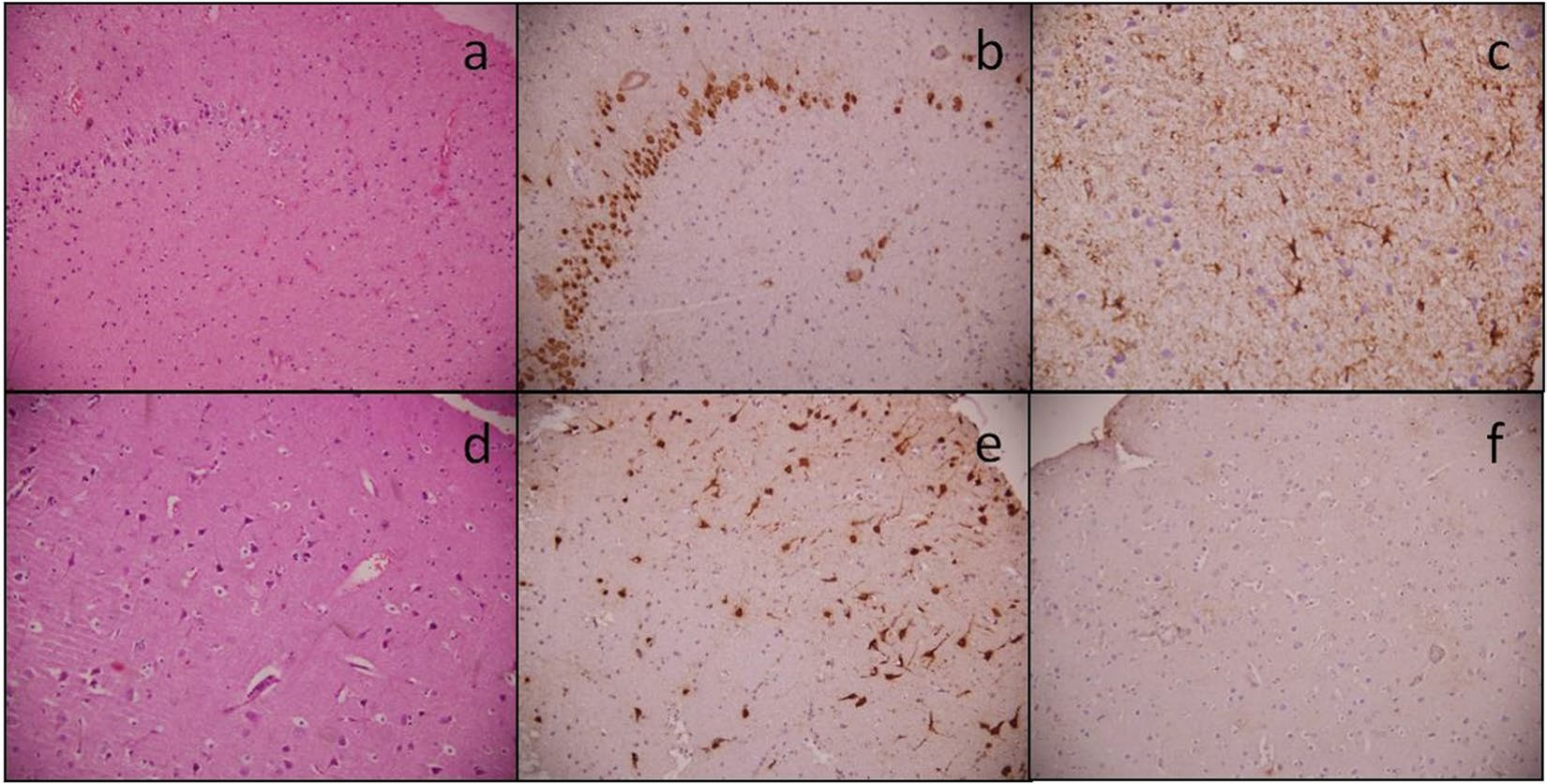
Photomicrographs showing histopathology of the hippocampus and anterior temporal lobe. Representative pictures of the hippocampal specimen showing loss of neurons (**a**) HE, x200) highlighted by Neu N (**b**) IHC, x200), while GFAP shows reactive gliosis (**c**) IHC, x400). Anterior temporal lobe specimens show no loss of neurons (**d**) HE, x200 and (**e**) Neu N IHC, x200) or reactive gliosis (**f**) GFAP IHC, x200).

The main pathological finding observed in excised tissue from patients with HS and treated with temporal lobectomy was confirmed by histopathology. Detailed histological examination of the tissues obtained from all the patients with HS (as mentioned in Table 1) were carried out. Typical aspects of a sclerotic hippocampus were observed in all the patients. We assessed for neuronal dropout leading to loss of normal laminar architecture, as highlighted on Neu N staining. Sections from the hippocampus showed dispersion of neurons as well as loss of neurons (Fig. 1a), as highlighted by NeuN staining (Fig. 1b). GFAP staining demonstrated reactive gliosis in the hippocampus (Fig. 1c). Sections from anterior temporal lobe (ATL) tissue showed the presence of ischemic neurons in few cases; however, no apparent neuronal loss was observed (Fig. 1d and e). Reactive gliosis was absent in the ATL samples as GFAP staining did not show any positively stained reactive astrocytes (Fig. 1f). These results suggested that structural abnormalities like, loss of neurons and gliosis, was primarily present in the hippocampus of patients with HS.

**Table 1:** Characteristics of patients selected for the study.

Patient ID*	Sex	Age (Years)	Durationof Epilepsy (Years)	Semiology	AEDs	ECoG grading^22, 23^^,^**
E1	M	40	38	Dyscognitive episodes with ictal aphasia	Carbamazepine, Lacosamide, Clobazam	ATL-4 H-3
E2	M	35	21	Episodes of unresponsiveness with oral and bimanual automatisms	Levetiracetam, Carbamazepine, Clobazam	ATL-3 H-5
E3	F	24	9	Dyscognitive episodes, sings, laughs and covers face; left upper limb immobile	Carbamazepine, Clobazam	ATL-4 H-4
E4	F	16	4	Left upper limb jerks with bicycling movements with both legs	Carbamazepine, Levetiracetam, Sodium valporate	ATL-3 H-3
E5	M	15	9	Unresponsiveness with oral automatisms, no speech	Clobazam, Sodium valproate, Phenobarbital	ATL-3 H-4
E6	F	36	22	Unresponsiveness with fisting & moving backward of left hand.	Oxcarbazepine, Levetiracetam, Clobazam	ATL-2 H-3
E7	F	23	5	Dyscognitive episodes with left upper and lower limb clonic movements and abduction.	Oxcarbazepine, Levetiracetam, Clobazam	EH-1 H-3
E8	M	15	2	Unresponsiveness with left upper limb dystonia and jerking.	Lacosamide, Clobazam, Lamotrigene, Levetiracetam	ATL-3 H-4
E9	M	36	20	Unresponsiveness with head going to right side, bimanual automatisms.	Carbamazepine Lacosamide, Levetiracetam	ATL-2 H-3
E10	M	9	1	Left upper limb jerks with bicycling movements with both legs	Phenytoin, Carbamazepine, Risperidonesyp	ATL-2 H-3
E11	F	27	25	Closes ears, right hand fisted	Clobazam, Levetiracetam, Phenytoin, Sodium valproate, Clonazepam	ATL-3 H-3
E12	M	6	5	Right upper and lower limb abduction then clonic movements and spread to the other side	Lacosamide, Clobazam, Levetiracetam	EH-3 H-3
E13	F	27	16	Unresponsiveness with rubbing of eyes during seizure.	Oxcarbazepine, Levetiracetam, Clobazam	EH-3 H-3
E14	M	22	15	Dyscognitive episodes with bimanual automatisms in the form of praying.	Lacosamide, Clobazam, Lamotrigene, Levetiracetam	EH-3 H-3
C1	F	50	NA	NA	No AEDs	Not Done
C2	M	75	NA	NA	No AEDs	Not Done
C3	M	38	NA	NA	No AEDs	Not Done
C4	M	30	NA	NA	No AEDs	Not Done
C5	F	63	NA	NA	No AEDs	Not Done
C6	M	22	NA	NA	No AEDs	Not Done
C7	F	2	NA	NA	No AEDs	Not Done
C8	M	57	NA	NA	No AEDs	Not Done
C9	F	54	NA	NA	No AEDs	Not Done
C10	F	45	NA	NA	No AEDs	Not Done
C11	M	30	NA	NA	No AEDs	Not Done
C12	M	40	NA	NA	No AEDs	Not Done
C13	F	30	NA	NA	No AEDs	Not Done
C14	M	48	NA	NA	No AEDs	Not Done
C15	M	35	NA	NA	No AEDs	Not Done
C16	M	53	NA	NA	No AEDs	Not Done
C17	F	30	NA	NA	No AEDs	Not Done
C18	M	22	NA	NA	No AEDs	Not Done
C19	M	65	NA	NA	No AEDs	Not Done
C20	F	35	NA	NA	No AEDs	Not Done

E1–E14 = Patients with HS.

C1–C20 = Patients with glioma (non-seizure controls).

*MRI and histo-pathology in all patients showed hippocampal sclerosis.

**EcoG = electro-corticography.

NA = Not applicable.

### Spontaneous postsynaptic activity in pyramidal neurons of slice preparations obtained from the hippocampal and ATL regions of patients with HS

**Figure 2:**
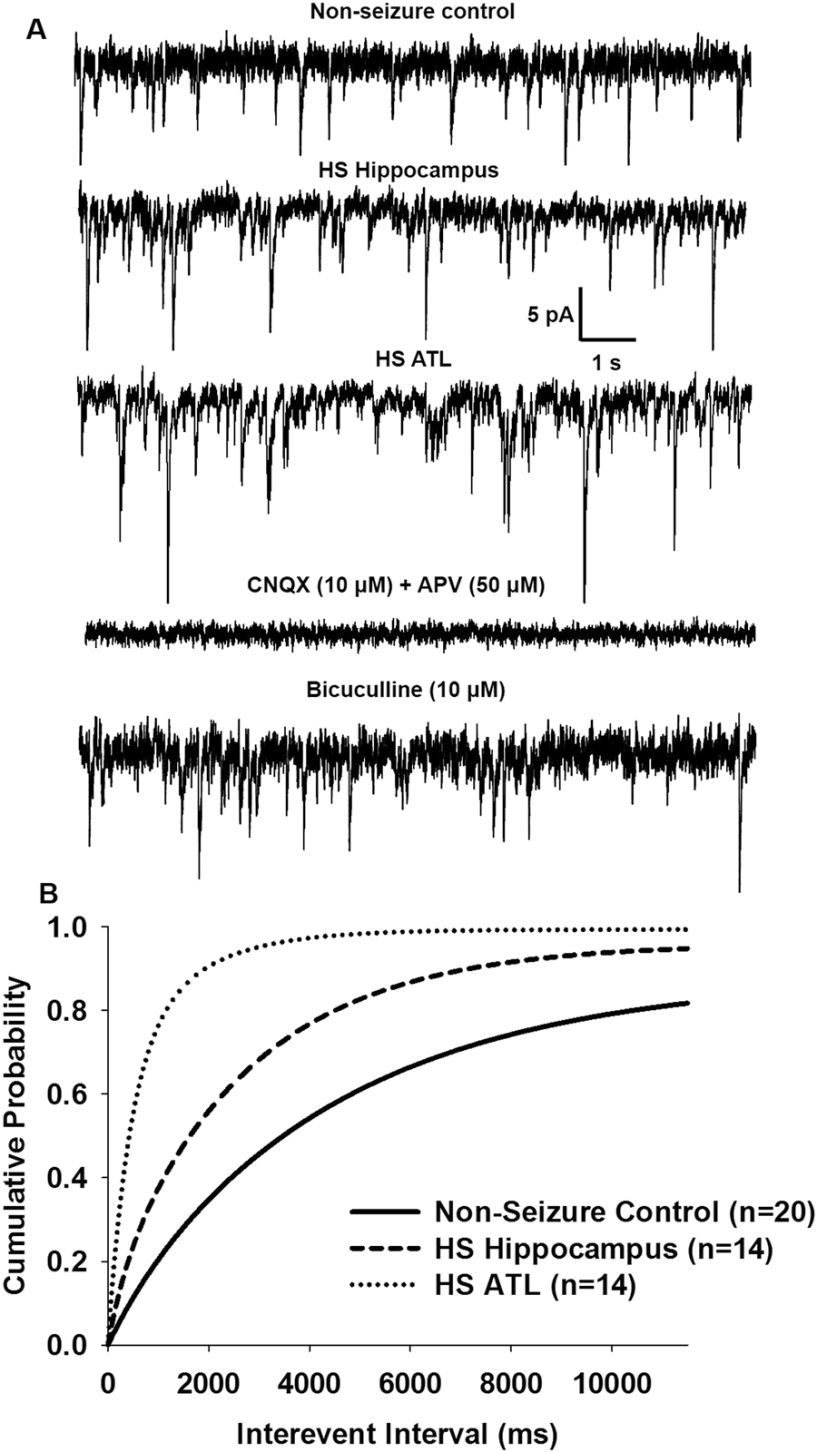
Spontaneous EPSCs in pyramidal neurons of resected brain samples. (**A**) Sample recordings of spontaneous EPSCs recorded from pyramidal neurons of resected brain samples obtained from non-epileptic control (top trace). The second trace shows spontaneous EPSCs recorded from pyramidal neurons of the hippocampal samples of patients with HS at an expanded time scale. Third trace shows recording from pyramidal neurons of the ATL samples of patients with HS. Fourth trace shows recording following superfusion of the HS hippocampal slice with glutamate receptor antagonists APV (50 μM) and CNQX (10 μM) for 10 min. Bottom trace shows recording following superfusion of the HS hippocampal slice with GABA_A_ receptor antagonist bicuculline (10 μM) for 10 min. (**B**) Cumulative probability plots of inter-event intervals of spontaneous EPSCs recorded from slice preparations of non-epileptic control and the hippocampal and ATL samples of HS patients. Plots represent data from twenty neurons from twenty patients for non-seizure control, eighteen neurons from the hippocampal samples of fourteen HS patients and sixteen neurons from the ATL samples of fourteen HS patients. Compared to non-seizure controls a significant leftward displacement of the cumulative distribution of inter-event intervals was observed in the hippocampal samples (p = 0.007 according to K-S test) and the ATL samples (p < 0.002 according to K-S test) of patients with HS.

Spontaneous EPSCs on pyramidal neurons were recorded at −70 mV under whole-cell voltage clamps in samples obtained from non-seizure controls (cortical samples obtained from the periphery of tumour of patients undergoing surgery for glioma) as well as hippocampal and anterior temporal lobe samples obtained from patients with HS. These currents, which appeared as inward events, were completely blocked following 10 min superfusion of the slices with ACSF containing the AMPA receptor antagonist CNQX (10 µM) in an admixture with the NMDA receptor antagonist APV (50 µM) (Fig. 2A). These results support the contention that these events are spontaneous EPSCs mediated by AMPA/kainite and NMDA receptors. The frequency of EPSCs remains unaffected by bath application of GABA_A_ receptor antagonist bicuculline (10 μM), suggesting that glutamatergic activity in the pyramidal neurons is not influenced by the basal GABAergic activity in the slices at −70 mV (Fig. 2A). Similarly, spontaneous EPSCs were also recorded from slice preparations obtained from the hippocampal and anterior temporal regions of patients with HS (Fig. 2A). Figure 2B shows that the normalized cumulative inter-event interval distributions in pyramidal neurons of the hippocampal (n = 14) and ATL samples (n = 14) were shifted to a higher interval in comparison with the non-seizure control (n = 20). The mean frequency of spontaneous EPSCs in the hippocampal samples was 0.99 ± 0.13 Hz, while in the case of samples obtained from the ATL, it was 1.39 ± 0.15 Hz. The mean amplitude of the spontaneous EPSCs in the hippocampal and ATL samples obtained from HS patients were significantly greater than the non-seizure controls (Table 2). However, the rise time and decay time constant (τ_d_) in HS samples were not significantly different from those in non-seizure controls (Table 2), which may be because spontaneous EPSCs were mostly recorded from the soma of pyramidal neurons. Bath application of an admixture of CNQX (10 µM) with APV (50 µM) completely blocked the glutamatergic events in both the hippocampal and ATL samples from patients with HS. Together, these findings suggested enhanced glutamatergic activity in both the hippocampal and ATL samples of HS, while the magnitude of increase was higher in the ATL samples compared to the hippocampal samples.

**Table 2:** Kinetics of Spontaneous EPSCs.

Slice Preparation	Non-seizure control (n = 20)	HS- Hippocampus (n = 14)	HS-ATL (n = 14)
Amplitude (pA)	12.52 ± 2.2	16.65 ± 2.0*	18.96 ± 2.0**
Rise Time (ms)	2.3 ± 0.6	2.8 ± 0.8	2.6 ± 0.7
Decay time constant, τ_d_ (ms)	10.6 ± 1.9	8.2 ± 1.2	10.1 ± 1.5

Characteristics of spontaneous EPSCs recorded from pyramidal neurons in resected samples from non-seizure control and the hippocampal and ATL samples from HS patients. The data are presented as the mean ± S.E.M. *p = 0.04; **p = 0.009 compared to non-seizure control according to one-way ANOVA followed by Tukey post hoc test.

**Figure 3:**
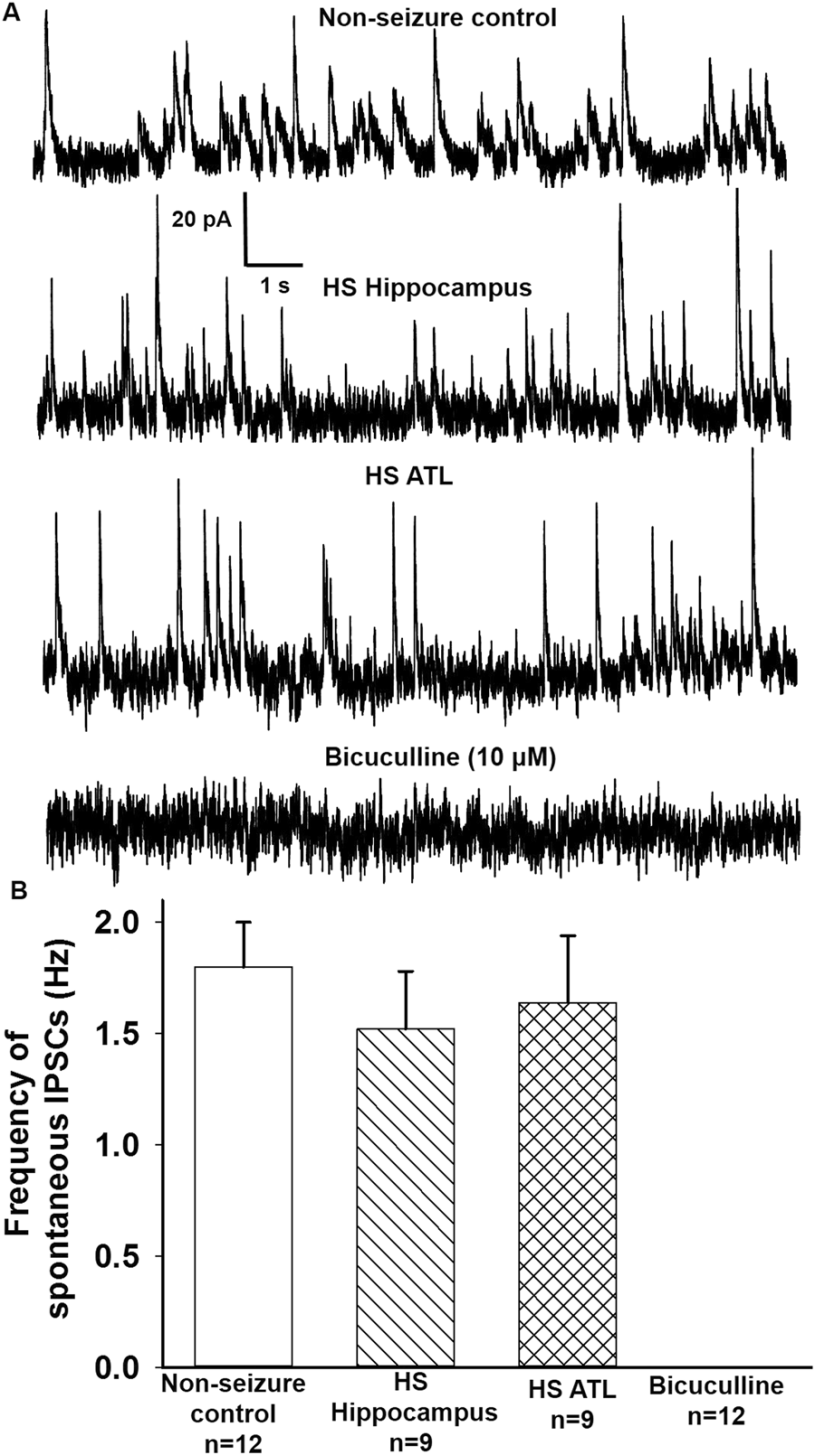
Spontaneous IPSCs from pyramidal neurons of resected brain samples. (**A**) Sample recordings of spontaneous GABAergic PSCs at 0 mV obtained from pyramidal neurons of non-seizure control, and the hippocampal and ATL samples obtained from patients with HS. Bottom trace shows recordings at 0 mV 10 min following superfusion of a HS hippocampal slice with ACSF containing GABA_A_ receptor antagonist bicuculline (10 μM). (**B**) Mean frequency of GABAergic PSCs recorded from (i) non-seizure controls, (ii) HS Hippocampal sample, (iii) HS ATL sample and, (iv) bicuculline treatment. Graph and error bars represent mean and S.E.M., respectively, of data obtained from twelve non-seizure control samples from twelve patients, and nine hippocampal and ATL samples obtained from nine patients with HS.

We also recorded the spontaneous inhibitory postsynaptic currents (IPSCs) at 0 mV from the pyramidal neurons of non-seizure control and the hippocampal and ATL samples of HS (Fig. 3A). The spontaneous IPSCs were completely blocked by superfusion of slices with ACSF containing bicuculline (10 μM). However, we did not observe a significant difference in the frequency of spontaneous inhibitory postsynaptic currents (IPSCs) recorded from the hippocampal and ATL samples of HS patients compared to non-seizure controls (Fig. 3B). We also observed that the mean peak amplitude of the spontaneous IPSCs in the hippocampal (22.2 ± 2.1 pA) and ATL (24.8 ± 1.8 pA) samples from patients with HS was similar to that in case of non-seizure controls (20.6 ± 0.93 pA).

### Suppression of spontaneous EPSCs in pyramidal neurons of slice preparations obtained from hippocampal and ATL samples by Na^+^-channel blocker tetrodotoxin (TTX)

**Figure 4:**
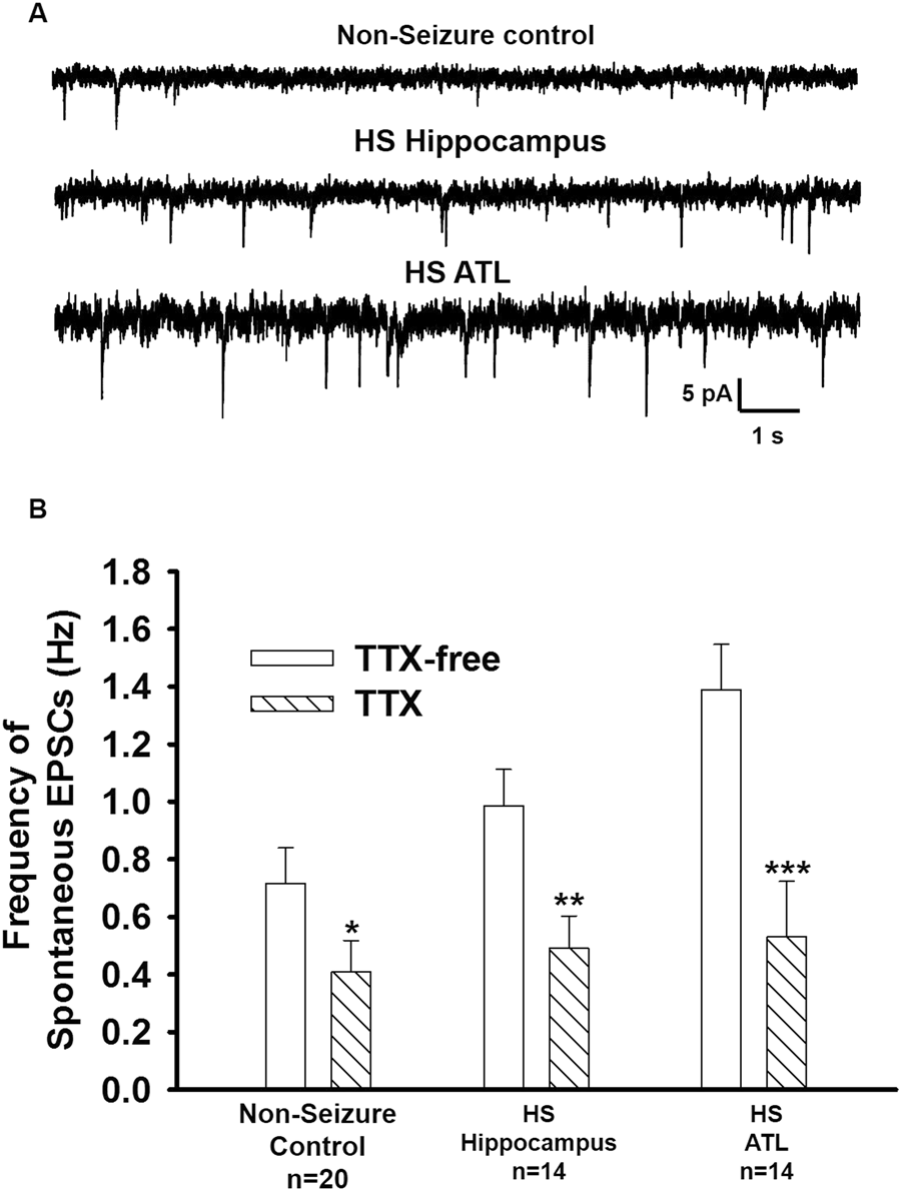
Effect of TTX on frequency of spontaneous EPSCs. (**A**) Sample recordings of spontaneous EPSCs recorded from pyramidal neurons of non-epileptic control and the hippocampal and ATL samples of HS after superfusion of slice with action-potential inhibitor TTX (200 nM) for 10 min. (**B**) Mean frequency of EPSCs in each of the above-mentioned samples before and after superfusion with ACSF-containing TTX (200 nM). Data were obtained from twenty neurons from twenty patients for non-seizure control, fourteen neurons from the hippocampal samples of fourteen HS patients and sixteen neurons from the ATL samples of fourteen HS patients. *p = 0.02; **p = 0.007; ***p = 0.0009 compared to respective controls according to one-way ANOVA followed by Dunnett post hoc test.

**Figure 5:**
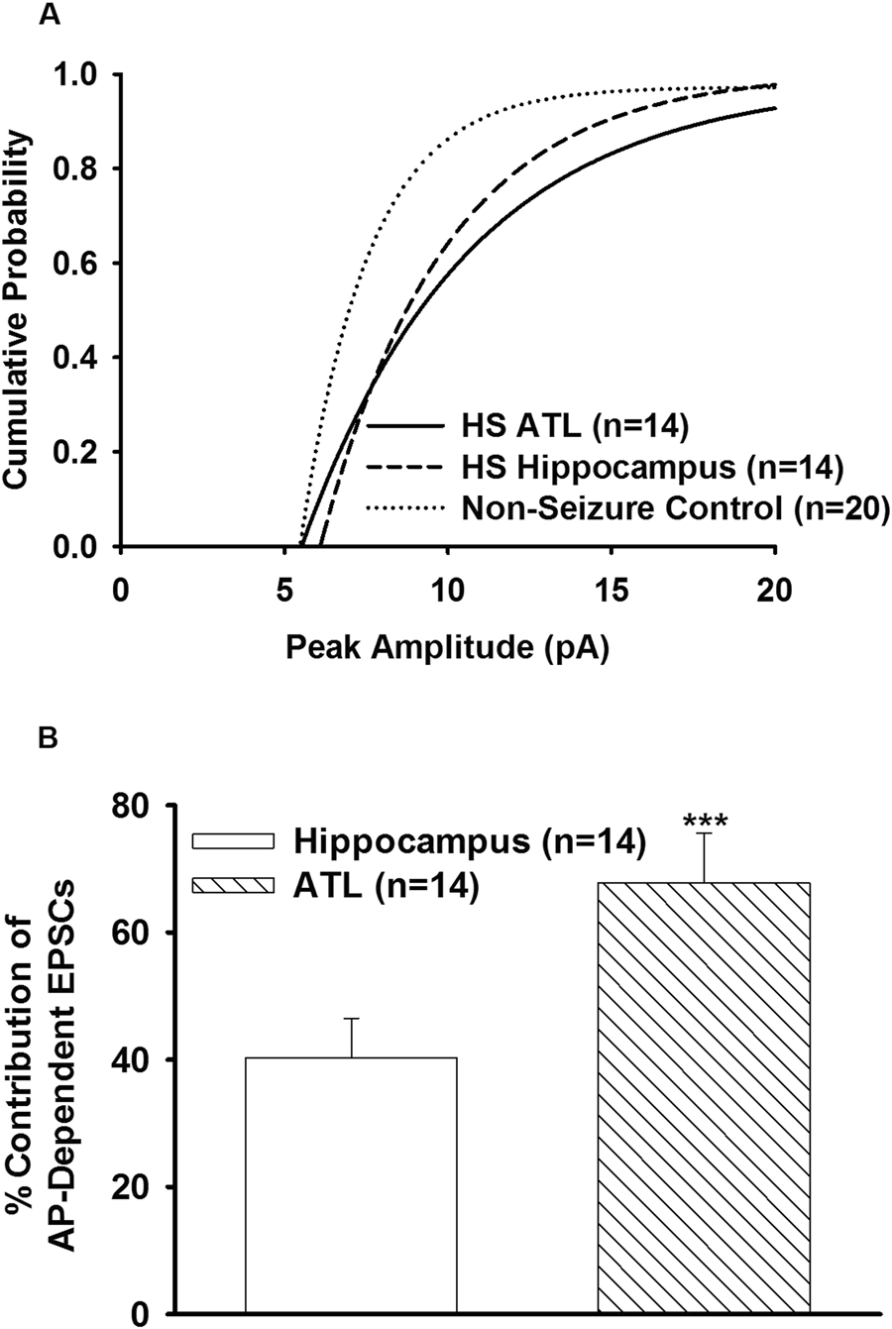
Contribution of action potential-dependent spontaneous EPSCs in the hippocampal and ATL samples. (**A**) Cumulative distribution of peak amplitude of mEPSCs recorded from non-epileptic control and the hippocampal and ATL samples of HS. In comparison to non-epileptic control, the cumulative distribution of peak amplitude of the hippocampal and ATL samples was displaced to the right (p = 0.03 for the hippocampal; p = 0.003 for ATL according to K-S test). (**B**) Graph shows the percent contribution of AP-dependent EPSCs in MTLE samples with respect to non-seizure control after bath application of TTX (200 nM, 10 min). Data were obtained from twenty neurons from twenty patients for non-seizure control, fourteen neurons from the hippocampal samples of fourteen HS patients and sixteen neurons from the ATL samples of fourteen HS patients. The magnitude of reduction in the frequency of spontaneous EPSCs caused by TTX in the ATL samples was significantly larger than that in case of the hippocampal samples. (***p = 0.0007 according to one-way ANOVA followed by Tukey post hoc test).

To isolate action potential (AP)-independent from AP-dependent glutamatergic EPSCs, slice preparations obtained from non-seizure controls as well as the hippocampal and anterior temporal lobe samples obtained from patients with HS were superfused with TTX (200 nM)-containing ACSF for 10 mins. TTX reduced the mean frequency and the mean amplitude of glutamatergic events by 43.9 ± 2.2% and 50.9 ± 2.3%, respectively, in the non-epileptic control (Fig. 4; Table 3). These results were consistent with TTX-induced block of the action potential-dependent glutamatergic transmission; only miniature EPSCs (mEPSCs) remained in the presence of TTX. In the hippocampal neurons of HS (n = 14), the frequency of spontaneous EPSCs in TTX-containing ACSF was reduced by 50.26 ± 2.1%, while in the ATL samples (n = 14) it was reduced by 61.8 ± 3.1% (Fig. 4). TTX reduced the mean amplitude of glutamatergic events by 24.7 ± 5.2% and 47.8 ± 7.6% in the hippocampal and anterior temporal lobe samples, respectively, compared to that in the case of the non-seizure control (Table 3). Application of TTX significantly reduced the mean amplitude of spontaneous EPSCs in all the samples as summarized in Table 3. Furthermore, we did not observe any significant difference in the frequencies of mEPSCs between the non-seizure control and the hippocampal and ATL samples obtained from patients with HS (Table 3). In the hippocampal neurons, the reduction in the mean amplitude by TTX was higher compared to the ATL neurons of the HS patients (p = 0.02) as depicted by the mean amplitudes of mEPSCs (Table 3). The cumulative distribution of mEPSC amplitudes was also skewed toward larger amplitudes in the ATL samples compared to the hippocampal samples (Fig. 5A). The rise time and decay time constant (τ_d_) of the mEPSCs were not significantly affected by TTX (Table 3). Furthermore, TTX blocked the action potential-dependent EPSCs in all the samples, and we found that the contribution of AP-dependent EPSC frequency with respect to non-seizure controls was significantly higher in ATL samples compared to hippocampal samples (Fig. 5B). Our data indicated that the increased glutamatergic tone in the hippocampal samples is different from that in the case of ATL samples of HS.

**Table 3:** Kinetic properties of miniature EPSCs (mEPSCs).

Slice Preparation	Non-seizure control	HS-Hippocampus	HS- ATL
	(n = 20)	(n = 14)	(n = 14)
Frequency (Hz)	0.41 ± 0.1	0.41 ± 0.1	0.53 ± 0.3
Amplitude (pA)	6.02 ± 1.6	8.95 ± 2.1*	9.92 ± 2.2**
Rise Time (ms)	2.1 ± 0.4	2.2 ± 0.6	2.5 ± 0.7
Decay time constant, τ_d_ (ms)	9.8 ± 1.5	9.2 ± 1.3	9.6 ± 1.4

Characteristics of mEPSCs recorded from pyramidal neurons in resected samples from non-seizure control and the hippocampal and ATL samples from HS patients in the presence of ACSF containing TTX (200 nM). The data are presented as the mean ± S.E.M. *p = 0.05; **p = 0.007 compared to non-seizure control according to one-way ANOVA followed by Tukey post hoc test.

### Differential regulation of mRNA and protein levels of NR2A and NR2B subunits of NMDA receptors in the hippocampal and ATL samples

**Figure 6:**
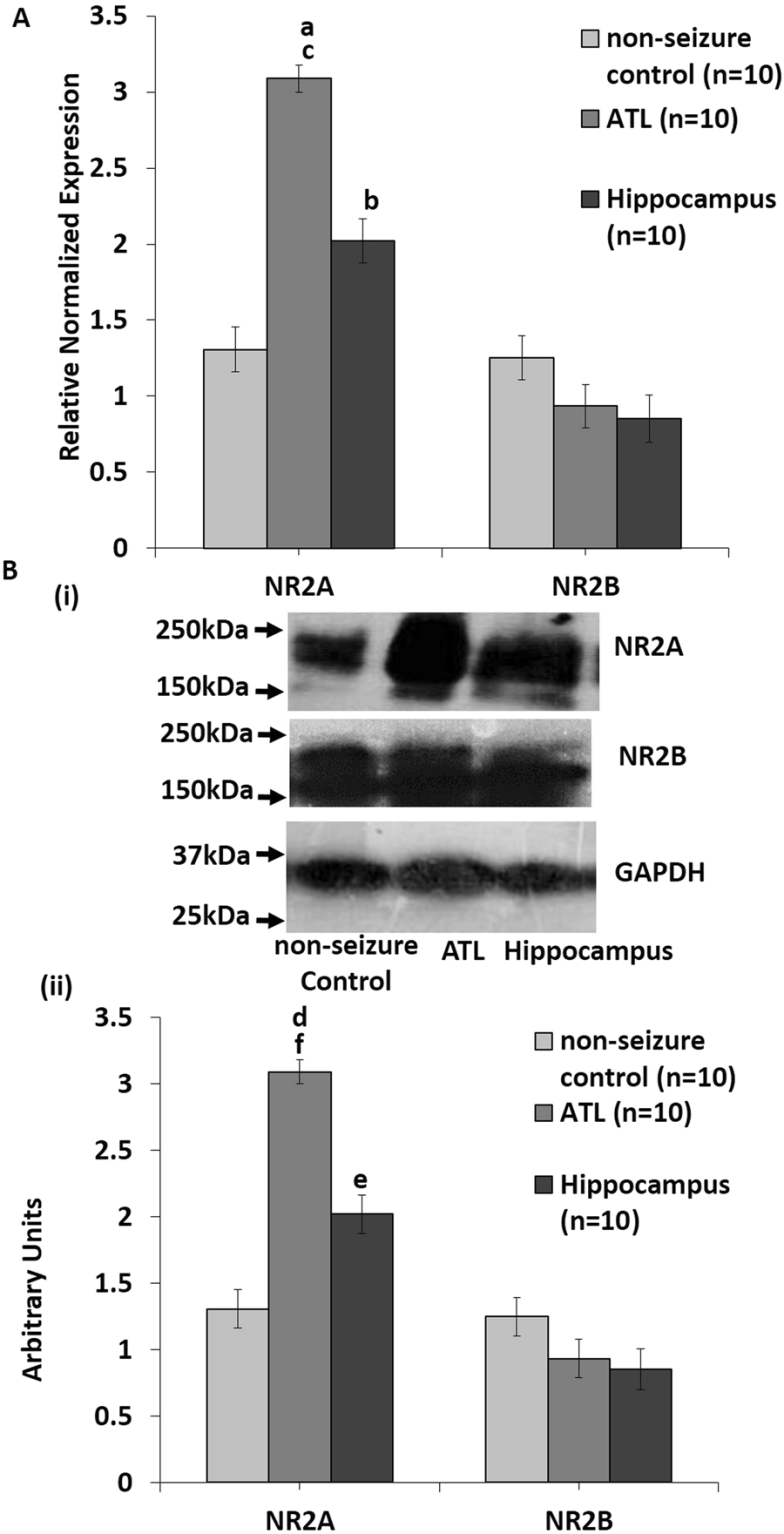
Increased expression of NR2A subunits might contribute differentially to the hyperexcitability in the ATL and the hippocampal regions (**A**). qPCR analysis showing increased NR2A and unaltered NR2B mRNA levels. Relative changes in gene expression were calculated using the ΔΔCq method with HPRT as a reference gene. Mean increase in NR2A transcripts in both ATL and the hippocampal regions were statistically significant (a: p = 0.003, b: p = 0.003) with respect to non-seizure control according to one-way ANOVA followed by Dunnett post hoc test, and also in ATL region with respect to the hippocampal region (c: p = 0.006) according to one-way ANOVA followed by Tukey post hoc test. No significant change was observed in the mRNA levels of NR2B gene in both ATL and the hippocampal regions. Error bar is ± SEM based on 10 patient and 10 control samples and each sample was analyzed in triplicates. (**B**) (i) Western blotting analysis of NR2A and NR2B in tissues resected from the ATL and the hippocampal regions. Blots show a single band at the predicted size of ~177 kDa for NR2A, ~166 kDa for NR2B and ~37 kDa for GAPDH. Representative immuno-blots from control and MTLE patients showing differentially increased expression of NR2A and unaltered NR2B levels in ATL and the hippocampal regions of MTLE patients as compared to the control. Molecular weight markers (kDa) are depicted to the left. (ii) The graph represents data from densitometric analysis of NR2A and NR2B in the ATL and the hippocampal samples of MTLE patients as compared to non-seizure control, by quantifying band intensities normalized to GAPDH for individual samples. Densitometries are expressed in arbitrary units (AU) and data is presented as ATL ± SE (n = 10), H ± SE (n = 10) specimens versus control ± SE (n = 10); statistical analysis using one-way ANOVA with Dunnett’s *post hoc* test d: p = 0.006, e: p = 0.043 with respect to non-seizure control using one-way ANOVA followed by Tukey’s post-hoc test f: p = 0.034, between ATL and the hippocampal regions.

Quantitative PCR was performed in triplicate to quantify the difference in the expression of NR2A and NR2B subunits in both the hippocampal (n = 10) and ATL (n = 10) samples resected from HS patients. As shown in Fig. 6A, a significant increase in the expression of NR2A was observed in both ATL (3.2-fold ± 0.087; p = 0.002) and hippocampal (2.08-fold ± 0.132; p = 0.003 according to one-way ANOVA followed by Dunnett post hoc test) tissues compared to non-seizure control, although the fold change was higher in the ATL tissues compared to the hippocampal tissues (p = 0.006 according to one-way ANOVA followed by Tukey post hoc test). No significant change was observed at the mRNA levels of the NR2B gene in both ATL (0.92-fold ± 0.135) and hippocampal (0.83-fold ± 0.146) tissues (Fig. 6A). Western blotting analyses of NR2A revealed significant upregulation of NR2A protein levels in both the ATL (p = 0.006) and the hippocampal samples (p = 0.043) compared to the control. Upregulation of NR2A in the ATL region was significantly higher than the hippocampal samples (p = 0.035). Again, no significant change was observed at the protein levels of the NR2B gene in both ATL and hippocampal tissues (Fig. 6B). Taken together, these results suggest differentially increased expression of the glutamate NMDA receptor NR2A subunit both at the mRNA and protein levels in the hippocampal and ATL regions, whereas NR2B expression is unaltered in both the regions in patients with HS.

### Ratio of frequency and peak amplitude of glutamatergic-GABAergic synaptic activity varied in the hippocampal and ATL samples

**Figure 7:**
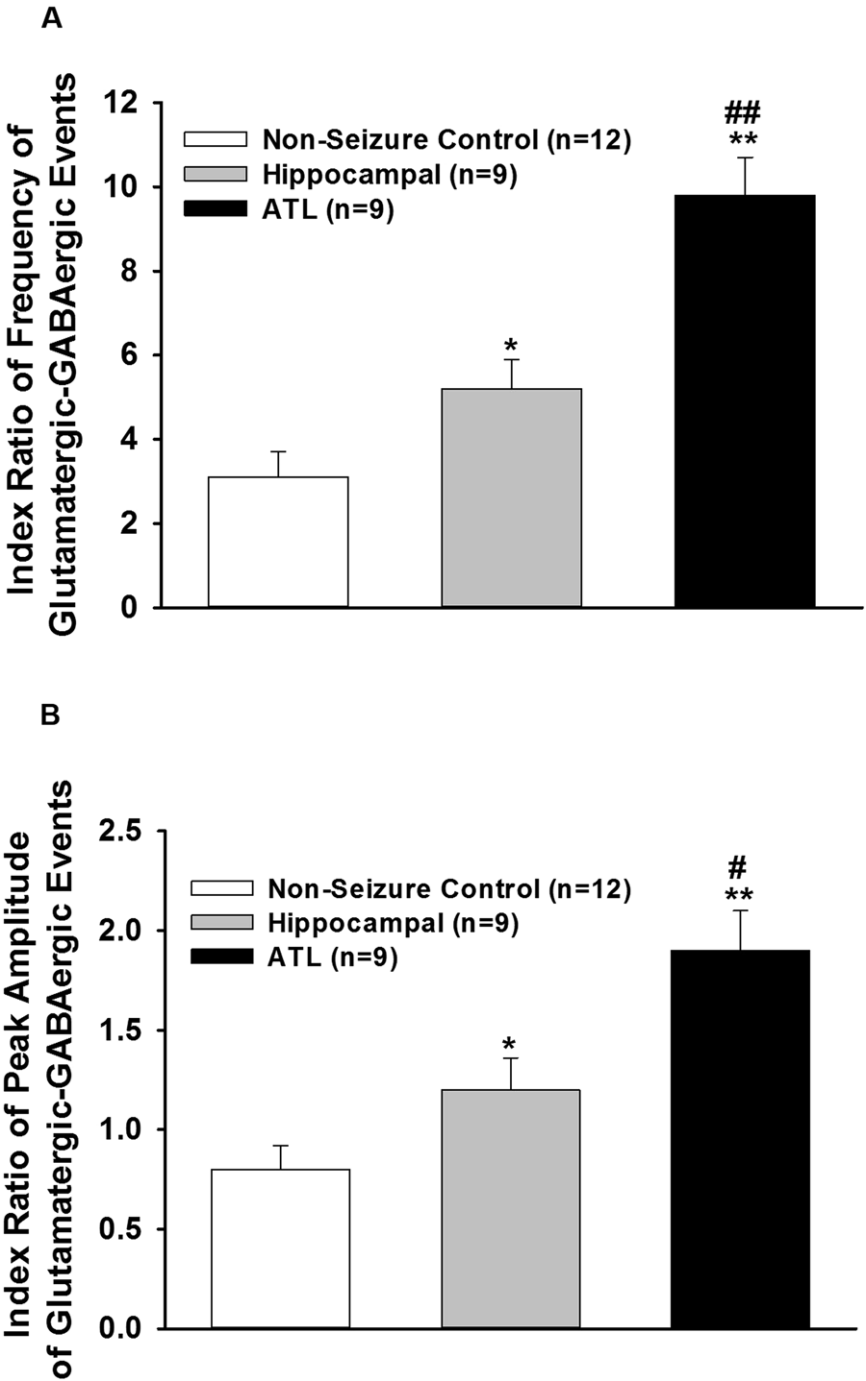
Index ratio of glutamatergic-GABAergic events. (**A**) Index ratio for frequency was obtained by dividing the average frequency of glutamatergic events by that of GABAergic events in a subset of pyramidal neurons where the spontaneous EPSCs and IPSCs could be recorded in the same cell of non-seizure control and the hippocampal and ATL samples of patients with HS. In comparison to the ATL samples the index ratio of frequency was lower in the hippocampal samples. (**B**) Index ratio for peak amplitude was obtained by dividing the mean peak amplitude of glutamatergic events by that of GABAergic events in a subset of pyramidal neurons where the spontaneous EPSCs and IPSCs could be recorded in the same neuron of non-seizure control and the hippocampal and ATL samples of patients with HS. The index ratio of peak amplitude in the hippocampal samples was lower compared to the ATL samples. Graph and error bars represent mean and S.E.M., respectively, of data obtained from twelve non-seizure control samples from twelve patients, and nine hippocampal and nine ATL samples obtained from nine patients with HS. *p < 0.05; **p < 0.001 compared to non-seizure control according to one-way ANOVA followed by Dunnett post-hoc test. ^#^p < 0.05; ^##^p < 0.01 according to one-way ANOVA followed by Tukey post hoc test.

Index ratios were obtained by dividing the average frequency and the mean peak amplitudes of glutamatergic events by that of GABAergic events in a subset of neurons where the glutamatergic and GABAergic synaptic activities could be recorded in the same cell. GABAergic inhibitory post-synaptic currents (IPSCs) were recorded at a holding potential of 0 mV (Fig. 3). Spontaneous IPSCs were observed as outward currents, which were abolished by the application of bicuculline (10 μM). This analysis included nine cells from the hippocampal region and eleven cells from the ATL region of HS patients. The ratio of frequency and peak amplitude of glutamatergic/GABAergic events were significantly higher in the hippocampal (n = 9) and the ATL samples (n = 9) obtained from the HS patients compared to the ratio in non-seizure controls (n = 12) as shown in Fig. 7. The average glutamatergic-GABAergic event frequency ratio in the hippocampal sample was lower (5.2 ± 0.7) than the ATL sample (9.8 ± 0.9; p = 0.006 according to one-way ANOVA followed by Tukey post hoc test) in these patients (Fig. 7A). The index ratio of glutamatergic-GABAergic event peak amplitude was also lower in the hippocampal samples (1.2 ± 0.16) compared to that in the ATL samples (1.9 ± 0.2; p = 0.04 according to one-way ANOVA followed by Tukey post hoc test) obtained from patients with HS. These data suggested that relative to inhibitory synaptic transmission, the excitatory synaptic transmission was higher in the ATL region compared to the hippocampal region of patients with HS.

## Discussion

The present results disclose evidence that supports the concepts that i) two resting-state networks are present in patients with HS, one arising from the hippocampus and the other from the anterior temporal lobe; and ii) the spontaneous glutamatergic tone varies in resected brain specimens obtained from these regions, potentially mediated by two independent cellular mechanisms. These concepts can help clarify complicated network reorganizations as depicted in patients with HS at the cellular level.

Under resting conditions, the frequency of the glutamatergic EPSCs recorded from pyramidal neurons in the hippocampal samples of HS patients were higher compared to the non-seizure controls^12^. Here, we showed that the magnitude of increase in the glutamatergic transmission in the ATL samples was significantly higher than that in the case of the hippocampal samples under resting conditions (Figs 2 and 4). This finding indicated that under resting conditions, the mechanism that leads to abnormal excitatory activity in these two regions was different. We found that under resting conditions, there was no significant effect of GABA_A_ receptor antagonist, bicuculline, on spontaneous EPSCs (Fig. 2), and there was no significant alteration of GABAergic synaptic transmission in both the hippocampal and ATL samples obtained from patients with HS (Fig. 3). This finding suggested that hyperexcitability in HS is regulated primarily by the prototypical glutamate inputs in the hippocampus and ATL. Two distinct types of spontaneous glutamatergic responses contribute to the excitatory transmission; one is the AP-independent spontaneous activity (mEPSCs), which reflects a quantal mechanism and is insensitive to the Na^+^ channel blocker tetrodotoxin (TTX). The other is an AP-dependent spontaneous release, which is mediated by an AP invading the presynaptic terminals. Compared with the spontaneous mEPSCs, these events are sensitive to TTX and are characterized by a greater amplitude, as they reflect a multi-bouton glutamate release that is triggered by the presynaptic AP. The result that Na^+^ blocker TTX reduced the frequency of spontaneous EPSCs in the hippocampal and ATL samples of HS patients as well as in non-seizure controls indicates an inhibition of the action potential-mediated events. The magnitude of contribution of the AP-dependent EPSC frequency to the increased glutamatergic activity on pyramidal neurons of ATL samples was higher than that in the case of the hippocampal samples (Fig. 5B). This finding indicated that under resting conditions, presynaptic glutamate release processes leading to increased excitatory activity in the hippocampus are different from those in the ATL region. The higher mean amplitude of mEPSCs in the ATL neurons compared to the hippocampal neurons of HS suggests that the increase in the release probability of glutamate in ATL neurons was higher compared to the hippocampal neurons in the HS samples. Because the normalized cumulative distribution is also suggestive of a shift toward larger amplitudes of mEPSCs (Fig. 5A) in the ATL samples relative to the hippocampal samples, the role of postsynaptic glutamate receptors cannot be ruled out. In addition, we found that the relative contribution of excitatory synaptic transmission and inhibitory synaptic transmission significantly varied in these two regions, further confirming the fact that under a resting state, aberrant synaptic activity in these two regions were different from each other. Thus, we speculated that the glutamatergic network reorganization responsible for hyperexcitability in the hippocampus is different from that in the case of ATL in patients with HS. Even though we observed neuronal loss and reactive gliosis in the hippocampal samples and no apparent loss of neurons and reactive gliosis in the ATL samples (Fig. 1), it is not possible to correlate the severity of damage with the hyperexcitability pattern in these two regions. The possible relationship between inflammatory reactions and hyperexcitability in the hippocampus and ATL needs to be explored in future.

Endogenous NMDA receptor activity plays a crucial role in the generation of enhanced excitatory activity in the hippocampal samples obtained from patients with HS^12^. Using RNA-seq analysis of the hippocampal tissue resected from patients with HS, we have previously identified hubs of genes linked to synaptic transmission and neuronal network modulation^18^. We reported significant alterations in the expression levels of transporters, receptors and molecules involved in calcium signalling^18^. However, in that study we could not detect mRNA level alterations of NR2A levels in the RNAseq analysis, which could be attributed to the small sample size. In the current study, we not only found differential upregulation of NR2A mRNA levels but also differentially increased protein levels in the hippocampal and the ATL samples. NMDARs with different subunit combinations differed in their electrophysiological properties as well as their sensitivities to modulation by intracellular messengers^19^. NR2A and NR2B subunits prevailed in the adult cortex and the hippocampus, and previous studies have proposed that an increase in the NR2A/NR2B ratio in response to enhanced neural activity may not only contribute to maturational differences in the human hippocampal NMDAR function but may also play a role in the pathophysiology of neurodevelopmental disorders^20, 21^. In this study, we examined the contribution of these two subunit-containing NMDARs to the hyperexcitability observed in the ATL and the hippocampal samples. Our findings suggest differential upregulation of the mRNA as well as protein levels of NR2A between the ATL and the hippocampal samples, while no significant change was observed in the NR2B levels (Fig. 6). This finding again highlights the fact that the mechanism of NMDA receptor-mediated hyperexcitability varies between the hippocampal and ATL regions of patients with HS. Previous reports on rat models of limbic epilepsy are also suggestive of a contribution of NR2A-containing NMDA receptors in epileptogenesis and the development of mossy fiber sprouting, whereas NR2B-containing NMDA receptors were not shown to contribute in epileptogenesis and mossy fiber sprouting^13^. We speculated that the differential expression of NR2A subunit-containing NMDARs might contribute to the distinct hyperexcitability pattern in the ATL and the hippocampal regions of patients with HS.

In conclusion, this is the first direct evidence that large-scale networks exist at the cellular level in patients with HS under resting conditions, further confirming the conjecture that HS is a distributed network disorder and not a focal disorder. We have shown that under resting state independent of spiking activities, patients with HS showed two aberrant networks: one emanating from the hippocampus and the other from the anterior temporal lobe. Our study suggests that the excitatory synaptic connections in these two regions varied significantly, thereby causing a difference in the glutamatergic tone. This finding indicated that the reinforced glutamatergic synaptic connectivity to form a network in the hippocampus was different from that in the ATL in patients with HS. Thus, an anterior temporal lobectomy along with amygdalo-hippocampectomy is likely to have a better outcome than selective amygdalo-hippocampectomy, which spares the anterior temporal lobe. This study also provided some interesting information on the complexity of networks that may exist at the cellular level in HS, which may partly explain the reduction in the overall seizure-free outcome on long-term follow-up in patients undergoing surgery for HS.

The main limitation of this study was the inability to sample extra-temporal tissues during surgery due to ethical considerations. In addition, the possibility of finding altered glutamatergic synaptic mechanisms in the extra-temporal regions cannot be ruled out. At this stage, it also cannot be ascertained whether the networks in ATL were initially dependent or linked to those in the hippocampus and later became independent due to kindling or were *de novo* in nature. Another limitation of this study was the use of margins of tumors from patients with gliomas without seizures as non-seizure controls. Ideally, the non-seizure control could be samples resected from the temporal lobe structures of patients with similar ages with non-epilepsy pathologies such as glioma. However, it is ethically not possible to obtain region-specific tissues, and most of the temporal lobe tumors are associated with seizures. The temporal lobe samples obtained from autopsy patients were also not suitable to perform cellular electrophysiological studies. Thus, region-specific alterations in glutamatergic tone observed in the current study needs to be confirmed in a relevant animal model of HS. Future studies are also needed to differentiate the role of NMDA receptors and AMPA receptors in the hippocampus and ATL of patients with HS. In addition, further investigations to differentiate the involvement of NR2A and NR2B-containing NMDARs should also be considered.

## Materials and Methods

### Subjects & clinical evaluation

Patients with HS underwent phased pre-surgical assessment, and the pathology in each patient was demonstrated by documenting convergent data on MRI, video EEG (vEEG), Fluoro-2-deoxyglucose positron emission tomography (FDG-PET) evaluations and electrocorticography (ECoG) and confirmed by histopathological examinations in accordance with the recent World Health Organization classification. Fourteen patients with HS who underwent surgery from September 2014 to December 2015 were included in this study (Table 1). Six of the patients were female. The inclusion criteria included MRI-vEEG-ECoG concordance, absence of any obvious of structural changes/pathologies in the extra-hippocampal regions (e.g., gliosis, atrophy), and absence of dual pathology (e.g., cortical dysplasia). The mean age of the patients was 23.6 ± 10.2 years. The duration of seizures averaged at 10.5 ± 8.3 months. The seizure onset was 13.1 ± 8.1 years. The average number of AEDs/patient was 3.1 ± 0.7. Surgery included a standard anterior temporal lobectomy along with amygdalo-hippocampectomy. The structures resected included anterior temporal lobe, amygdala, hippocampus up to the level of superior colliculus, uncus, para-hippocampal gyrus, enterorhinal cortex, and subiculum. Prior to resection, electrocorticography (EcoG) was performed by placing a 4 × 5 grid over the lateral surface of temporal lobe, and grading was performed under anesthesia conditions as previously described^22, 23^ (Table 1). All these patients were seizure-free post-operatively (Class I Engel outcome)^24^. Part of the excised tissue from the hippocampus and ATL was fixed in 10% neutral buffered formalin, processed and embedded in paraffin for immunostaining. Haematoxylin and eosin stained sections of the hippocampal and ATL were examined. Immunohistochemistry was performed on the formalin-fixed paraffin-embedded tissue using primary antibodies against NeuN (neuronal nuclear protein; 1:100; Dako, Denmark) and glial fibrillary acidic protein (GFAP; 1:800; Dako, Denmark), as described previously^25^. The experiments described in this study were performed per the guidelines of the institutional ethics committee (IEC), All India Institute of Medical Sciences, New Delhi and human ethics committee, National Brain Research Centre, Manesar. Informed consent was obtained from all patients and their family members prior to the start of the study. Research material for this study consisted of tissues from the hippocampus and anterior temporal region, which was a part of the planned surgical resection (hence had no additional risk for the patient). Patients with HS were treated with polytherapy and anti-epileptic drugs (AEDs) that included valproic acid, carbamazepine, phenytoin and levetiracetam. Non-seizure controls included cortical tissues obtained from the margins of tumors during surgical resection (again a part of the planned surgical resection) in patients with gliomas without seizures (Table 1). The experiments performed in the study were approved by IEC, All India Institute of Medical Sciences, New Delhi and human ethics committee, National Brain Research Centre, Manesar.

### Cellular electrophysiology

Hippocampal and anterior temporal lobe samples resected during surgery were immediately transferred to well carbogenated, ice-cold artificial cerebrospinal fluid (ACSF). ACSF consisted of CaCl_2_, 2 mM; NaHCO_3_, 25 mM; NaH_2_PO_4_, 1.25 mM; NaCl, 125 mM; KCL, 2.5 mM; MgCl_2_, 1 mM; and Glucose, 25 mM. Samples were further processed for cellular electrophysiological experiments as per the protocol reported earlier^12^. Briefly, samples were placed in well carbogenated, ice-cold ACSF within 2 minutes of resection. Within 15 minutes of resection, slices (350-µm thick) were prepared using a vibrating blade microtome. Slices were transferred to the recording chamber and perfused with carbogenated ACSF at the rate of 2 ml/min. Whole-cell recordings were obtained from the soma of visually identified pyramidal neurons in slice preparations according to the standard patch-clamp technique using an Axopatch 200B amplifier (Molecular Devices, USA). We have specifically chosen neurons which had a pyramid-like soma and a single thick tapering apical dendrite, as visualised using IR-DIC videomicroscopy, for our studies. Patch pipettes were filled with internal solution containing HEPES, 10 mM; Cs-methanesulfonate, 130 mM; EGTA, 10 mM; CsCl, 10 mM; MgCl_2_, 2 mM (pH adjusted to 7.3 with CsOH; 340 mOsm). Pipette resistance ranged between 3 and 5 MΩ. To record spontaneous EPSCs from neurons under whole-cell configuration at a holding potential of −70 mV, 5 mM QX-314 was added in the internal pipette solution. Spontaneous IPSCs were recorded at 0 mV. All experiments were performed at room temperature (~22 °C). Experiments on non-seizure control samples were performed in the same manner as in the case of HS samples.

EPSCs and IPSCs were analyzed using pCLAMP 10.0 software (Molecular Devices, Sunnyvale, USA). Visual inspection of each recording was performed to select single events with a sharp rising phase and an exponential decay for kinetic analysis of the EPSC events. The frequency, peak amplitude, rise time (10–90%), and decay-time constant (τ_d_) of the synaptic events were measured using the clampfit module of pCLAMP 10.0 software. EPSCs and IPSCs showing double- and multiple-peaks were excluded from analysis of kinetic properties but were utilized for the calculation of frequency of events as multiple events. The data were expressed as the mean ± S.E.M. of results obtained from various patients. Statistical tests included one-way ANOVA in Sigma plot 12.0 (Systat Software, Inc., Chicago, IL) to determine any significance in the data. Furthermore, the cumulative distributions of events in non-seizure control versus the hippocampal and ATL groups were compared using the Kolmogorov-Smirnov test (K-S test). For this, events from different neurons in each group were pooled together and then subjected to the K-S test using the Clampfit module of the pCLAMP10.0 software.

### Molecular biology studies

#### Tissue preparation

Approximately 5-mm-thick hippocampal and ATL samples resected during surgery from patients with HS were stored in RNAlater stabilization reagent (Qiagen) in order to prevent degradation of RNA for RNA extraction and snap frozen for western blotting analyses. All samples were stored at −70 °C until further processing. The samples with RNAlater were stored at 4 °C overnight and subsequently frozen and kept at −70 °C.

#### Quantitative real time PCR (qPCR)

Quantitative real-time polymerase chain reaction (qPCR) was performed to evaluate the expression of NR2A and NR2B subunits in the ATL and hippocampal regions Specific primers for selected NR2A, NR2B and HPRT were designed using the Primer-BLAST (Primer3Input, version 0.4.0 and BLAST, available at http://www.ncbi.nlm.nih.gov/tools/primer-blast/). NR2A-forward, 5′-CACGGAGAGAAACATTCGGAATA-3′, NR2A-reverse, 5′-AGACTGCGGCATCGTAGATGA-3′; NR2B-forward, 5′-AAGCCCCATCATTCTTCTTTACTG-3′, NR2B-reverse, 5′-CGATCCACGTGTAGCCATAGC-3′; HPRT-forward, 5′-F-GCTTTCCTTGGTCAGGCAGTA-3′, HPRT-reverse, 5′-GGTCCTTTTCACCAGCAAGCT-3′. qPCR was performed in an independent set of 10 patients (both hippocampal and ATL) and 10 non-seizure control samples. RNA was extracted, purified and analyzed for purity by calculating the RIN values as previously described^18^. Purified RNA was reverse transcribed using high capacity cDNA reverse transcription kit (Invitrogen, Carlsbad, CA, USA) following the manufacturer protocol. Real time PCR amplifications were performed in CFX96 Real Time System (Bio-Rad, USA) with the following cycling parameters: an initial hot start of 95 °C for 3 min followed by 40 cycles of 95 °C for 5 s and 60 °C for 30 s. To normalize qPCR reactions, HPRT (hypoxanthine phosphoribosyl-transferase) was included as a reference gene^18^. The 2^−ΔΔCq^ (Livak) Method (Bio-Rad CFX Manager software) was used to quantify the relative normalized expression of studied genes. Statistical significance was determined using one-way ANOVA comparing non-seizure control versus patient mean values.

#### Western blotting analysis

Frozen brain tissues were homogenized on ice in RIPA buffer (20 mM Tris-HCl, 150 mM NaCl, 1 mM Na2 EDTA, 1 mM EGTA, 1% NP-40, 1% sodium deoxycholate, pH 7.5), supplemented with protease inhibitor cocktail and phosphatase inhibitors. Protein concentrations were measured using a bicinchoninic acid Protein Assay Kit (Pierce, Rockford, IL, USA) and a microplate reader at 570 nm (microplate reader (iMark^™^ Microplate Absorbance Reader, Bio-Rad, USA), applied at concentrations of 25 µg protein per lane on 10% polyacrylamide gels along with precision plus protein dual color standards (Bio-Rad, USA) and transferred onto polyvinylidene fluoride membranes using standard procedures. Because NR2A (~177 kDa)/NR2B (~166 kDa) and GAPDH (~37 kDa) have large molecular weight differences, the same membrane was cut into two halves from the center and used for further development with the respective antibodies. GAPDH was used as a loading control. Briefly, the membranes were incubated overnight in primary antibody against NR2A (sc-390094; Santa Cruz Biotechnology, Inc.), NR2B (ab28373; Abcam) and GAPDH (sc-365062; Santa Cruz Biotechnology, Inc.) in 1:500 dilution in 5% skimmed milk in Tris buffered saline (200 mmol/L Tris-HCl, 1·37 mol/L sodium chloride, pH 7·6) with 0·1% Tween 20. Membranes were blotted using anti-mouse IgG-HRP (sc-2005) and anti-rabbit IgG-HRP (sc-2030) (Santa Cruz Biotechnology, Inc.) and images were detected with a FluorChem M imager (Cell BioSciences, Santa Clara, CA, USA) using enhanced chemilluminescence with Pierce Super Signal (Pierce Biotechnology; Rockford, IL, USA). Densitometry analysis of the desired bands in western blots was performed using Quantity One software (Bio-Rad, USA). All densitometries are expressed in arbitrary units (AU). The signal intensity of the loading control (GAPDH) was used for normalization. Statistical analysis was performed using one-way analysis of variance (ANOVA), post hoc Dunnett for comparison with non-seizure control, and post hoc Tukey for comparison between ATL with H samples using Sigma Plot 12.0 software. The data are presented as the mean ± SEM. A *p-*value of <0.05 was considered statistically significant.
